# Feasibility and acceptability of a multiple risk factor intervention: The Step Up randomized pilot trial

**DOI:** 10.1186/1471-2458-11-167

**Published:** 2011-03-17

**Authors:** Jennifer B McClure, Sheryl L Catz, Evette J Ludman, Julie Richards, Karin Riggs, Lou Grothaus

**Affiliations:** 1 Group Health Research Institute, 1730 Minor Ave., Suite 1600, Seattle, WA, USA

## Abstract

**Background:**

Interventions are needed which can successfully modify more than one disease risk factor at a time, but much remains to be learned about the acceptability, feasibility, and effectiveness of multiple risk factor (MRF) interventions. To address these issues and inform future intervention development, we conducted a randomized pilot trial (n = 52). This study was designed to assess the feasibility and acceptability of the Step Up program, a MRF cognitive-behavioral program designed to improve participants' mental and physical well-being by reducing depressive symptoms, promoting smoking cessation, and increasing physical activity.

**Methods:**

Participants were recruited from a large health care organization and randomized to receive usual care treatment for depression, smoking, and physical activity promotion or the phone-based Step Up counseling program plus usual care. Participants were assessed at baseline, three and six months.

**Results:**

The intervention was acceptable to participants and feasible to offer within a healthcare system. The pilot also offered important insights into the optimal design of a MRF program. While not powered to detect clinically significant outcomes, changes in target behaviors indicated positive trends at six month follow-up and statistically significant improvement was also observed for depression. Significantly more experimental participants reported a clinically significant improvement (50% reduction) in their baseline depression score at four months (54% vs. 26%, OR = 3.35, 95% CI [1.01- 12.10], *p *= 0.05) and 6 months (52% vs. 13%, OR = 7.27, 95% CI [1.85 - 37.30], *p *= 0.004)

**Conclusions:**

Overall, results suggest the Step Up program warrants additional research, although some program enhancements may be beneficial. Key lessons learned from this research are shared to promote the understanding of others working in this field.

**Trial registration:**

The trial is registered with ClinicalTrials.gov (NCT00644995).

## Background

It is well known that most major chronic diseases are caused by more than one behavioral risk factor, and risk behaviors tend to co-occur. In fact, most US adults meet criteria for more than one disease risk factor [1]. As the number of concurrent risk factors increase, risk of death increases and survival rates decrease [2]. Changing multiple risk factors can reduce health care costs substantially. Longitudinal data indicate that effectively treating two behaviors reduces costs by about $2000 per year [3]. Despite this, most interventions to promote health and reduce risk focus on single behavioral or mental health risk factors at a time. They do not target multiple factors. There is a pressing need for interventions which can effectively modify more than one risk factor, but large gaps remain in our knowledge about how best to design, implement, and evaluate these programs [4-8]. For example, will the public be receptive to these programs, which may be more intensive or viewed as more difficult because they focus on multiple behaviors? Is it better to target behaviors concurrently or sequentially? Is it better to target people already motivated to change or those not particularly interested in changing their behavior? Is it feasible to address mental distress symptoms and behavioral outcomes in tandem? These are just a few of the important questions to which we do not yet have good answers. In short, further research is needed to inform the feasibility and design of multiple behavioral risk factor interventions in order to move this field forward.

The current paper reports findings from a randomized pilot feasibility trial of a multiple risk factor telephonic intervention. The intervention targeted participants with moderate or greater depressive symptoms who also smoked and reported low to moderate physical activity levels. These target behaviors were chosen because they frequently co-occur. Depression affects approximately 10% of adults in the U.S. each year and it is estimated that 17% of adults will suffer from a major depressive episode during their lifetime [9,10]. In clinical smoking cessation trials, as many as 35% to 60% of smokers report a positive history of depression [11-14]. There is also a higher prevalence of current smoking among people with depression. While approximately 21% of adults in the U.S. smoke [15], in a recent population-based survey 43% of adults with depression were smokers [16]. Having a history of depression also places smokers at greater risk of recurrent major depression after quitting [17] and relapse to smoking [18-20]. Thus, there appears to be a significant inter-relationship between depression and smoking. In fact, depressed smokers were recently singled out as a special population that warrants culturally adapted smoking cessation treatment programs specifically tailored to meet their unique needs [21]. More research in this area is called for.

Smokers [22,23] and persons with depression [24] are also less likely to meet recommendations for regular physical activity. Even after controlling for obesity level, people who report moderate or greater depressive symptoms are less likely to engage in moderate or vigorous physical activity than those with no or low depressive symptoms [25].

Each of these risk factors - depression, smoking, and low levels of physical activity - is associated with the onset or exacerbation of numerous chronic health conditions (e.g., cardiovascular disease, stroke, cancer, diabetes, obesity). In short, these behaviors represent a tri-fecta of disease risk. As such, addressing these behaviors in a single intervention is a logical intervention goal, and success may be aided by potential synergistic effects between these behaviors. That is, changing one's behavior in one of these areas could plausibly lead to changes in the others. As depression lifts, people may have the energy and motivation to be more physically active or to quit smoking. Physical activity has also been shown to both improve mood [9,26]; reduce acute desire to smoke [27-29], and nicotine cravings and withdrawal symptoms [27,29-31]; and to even be related to smoking abstinence [32], particularly at higher activity levels [33-35]. Finally, if people quit smoking, they may have more energy to be physically active.

This research was designed to assess the feasibility and acceptability of the Step Up Wellness Program, a cognitive-behavioral MRF intervention program. The pilot trial was not intended to test the effectiveness of the intervention, rather to inform the merit of the intervention concept and guide design enhancements, which could be tested in a future randomized effectiveness trial. We report on the main outcomes of the pilot trial and share some key lessons learned so that others interested in this area can benefit from this knowledge as they tackle the challenge of creating effective multiple risk behavior intervention programs.

## Methods

### Setting, Participants, and Recruitment

**Figure 1:**
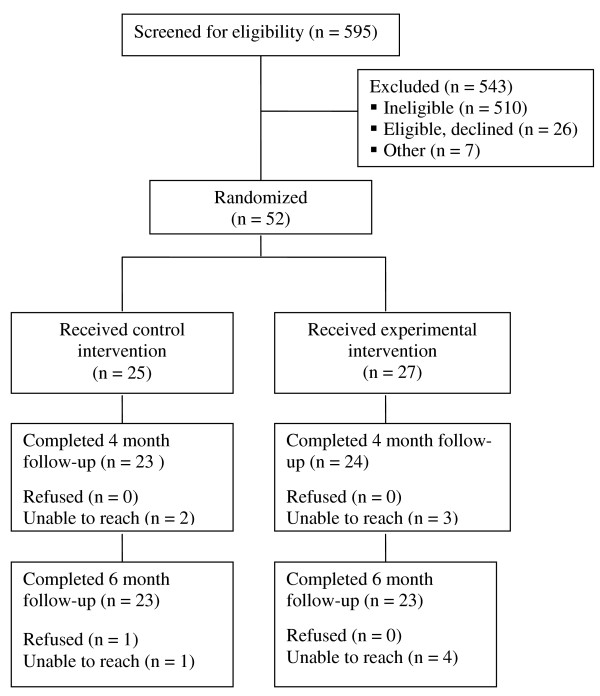
**Screening and recruitment**. Note: Participant flow.

Participants were recruited from the membership of Group Health, a large regional health plan in the Pacific Northwest. The recruitment flow is described in Figure 1. Potential participants were identified from automated medical records based on evidence of smoking in the past year and a depression diagnosis in the past two years, but no evidence of specialty mental health treatment in the prior year. Potential participants were mailed study invitation letters and then contacted by phone to be screened for interest and eligibility.

Participants were eligible if they were 18 to 75 years of age, currently enrolled in the health plan, endorsed at least moderate depressive symptoms (≥ 10) on the Patient Health Questionnaire (PHQ-9) [36,37] but were not currently being treated for depression by a licensed mental health provider, were a current smoker (regardless of interest in quitting), had a phone, were able to read and speak English, and did not self-report any mobility or physical activity impairments which would prevent participation in any aspects of the intervention. Participants also had to either fail to meet the current CDC guidelines for physical activity (i.e., 30 minutes of moderate activity five times a week or 20 minutes of vigorous activity three times a week) or meet criteria for low to moderate physical activity as assessed by the International Physical Activity Questionnaire (IPAQ) short-form [38]. The physical activity criteria were changed from the CDC guidelines to the IPAQ criteria during the course of the pilot because too few people failed to meet the CDC criteria based on their self-report. The IPAQ allowed us to screen out the most physically active individuals, while still retaining people who needed to either increase or maintain their activity level. Participants were excluded if they reported a diagnosis of schizophrenia or bipolar disorder or were taking an antidepressant medication for less than 30 days. Participants taking medications for more than one month who still showed signs of significant depressive symptoms were eligible because additional intervention appeared warranted; however, those taking a medication for less than 30 days did not have sufficient time for their depression to respond to pharmacological treatment.

Eligibility screening was done by phone. Eligible participants then attended an in-person meeting during which they provided written informed consent and completed a baseline interview. All subsequent contact occurred by phone. The primary reasons for being ineligible were: did not meet criteria for moderate or greater depressive symptoms (n = 261), reported being too physically active (n = 261), or did not smoke (n = 64). These exclusions were not mutually exclusive.

Pilot enrollment began in 2008 and ended in 2009. Data collection was completed in April, 2010. All activities were reviewed and approved by the Group Health Institutional Review Board.

### Intervention

Participants were randomly assigned to either usual care (n = 25) or the experimental intervention (n = 27), stratified by baseline antidepressant use. Usual care participants received standard self-help and information materials on depression, physical activity, and smoking developed and distributed by the health plan. They also received referral information for behavioral health services and the health plan's phone-based smoking cessation program. These materials were also provided to the experimental arm, but in addition participants in this group received a pedometer, Step Up program workbook, and a series of counseling calls from a study counselor. The counseling protocol included an initial motivational call designed to build program engagement, nine weekly cognitive-behavioral counseling calls during which participants were stepped through content in the program workbook, and two follow-up "booster" calls scheduled at flexible intervals based on participant need. Calls not completed before the six month follow-up assessment were not conducted. Each call was designed to last approximately 30 minutes and was audio-recorded. A random sample (~10%) was reviewed for treatment fidelity. The intervention was delivered by two masters-trained therapists, who were supervised by three doctoral-level clinical psychologists.

All intervention was delivered by phone. We had originally intended to pair the counseling with a weekly walking group; however, in a one arm pre-pilot trial (n = 10) conducted to fine tune the intervention materials, no participants attended the walking groups. Walking with strangers was reportedly a significant barrier to participation, so the group was dropped before the randomized pilot trial.

The intervention content was based largely on two prior phone-based treatment programs, one shown to be effective at improving depressive symptomology [39] and one shown to motivate smoking cessation [40]. We dropped the medication management focus of the original depression intervention and modified the counseling content and structure of the original programs to accommodate an integrated focus on mood management, building and supporting motivation for smoking cessation, and increasing physical activity using standard cognitive-behavioral therapeutic techniques (e.g., self-monitoring, goal setting, stimulus control, cognitive restructuring, stress management, and social support). The general content structure emphasized behavioral activation during the early counseling sessions, followed by cognitive restructuring in the latter sessions. Within each weekly call, however, the topics of mood, physical activity, and smoking were interwoven and presented in a way to help participants understand the inter-relationship between these behaviors and the potential synergistic effects of each behavior change. Participants were also assigned weekly homework 'experiments' to try. Experiments included cognitive restructuring exercises for mood management, behavioral activation exercises focused on increasing physical activity, and behavioral exercises designed to build self-efficacy for smoking cessation (e.g., learning to delay smoking in response to urges, practice quit attempts).

The program was presented as a wellness intervention designed to promote mental and physical well-being. Participants did not have to be ready to take action on any of the three target behaviors in order to be eligible for the study, just willing to talk about these issues each week with the counselor and agreeable in principle to trying the weekly experiments. In this sense, the intervention was designed to address each behavior concurrently; however, each week participants had the option of choosing which behavior(s) they were most interested in changing and which experiments they wanted to complete. Thus, in practice, attempts at behavior change were concurrent for some and sequential for others.

### Assessment

Follow-up assessments were conducted at four and six months post-enrollment. Interviewers were masked to participants' treatment assignment. Outcome measures included the Hopkins Symptom Checklist Depression Scale (SCL-D; 20 item) [41,42] for depressive symptoms, the proportion of individuals who reported a serious, 24-hour smoking quit attempt following treatment initiation, number of cigarettes per day, self-reported 7-day point prevalent abstinence (with non-responders coded as smoking), weekly duration (minutes) of moderate and vigorous physical activity assessed via the IPAQ short form [38], and days per week participants walked for exercise. In addition, baseline measures included demographics, stage of change for smoking cessation [43,44], motivation for changing each of the three outcome behaviors (depression, smoking, and physical activity) and self-efficacy for changing each outcome behavior. Motivation and self-efficacy were each assessed using Likert scale items ranging from 1 (not at all) to 10 (extremely).

At six month follow-up, intervention participants were asked to rate how helpful each component of the Step Up program was and how satisfied they were with the calls on a five point Likert scale from 1 (not at all) to 5 (extremely). In addition, we monitored participation in the counseling program, including the number of counseling calls completed and the duration of each call.

### Statistical Analyses

Since the primary purpose of this pilot was to assess the feasibility of the Step Up intervention, our primary outcomes were metrics of treatment participation, program satisfaction, and the helpfulness of each Step Up intervention component. We also compared treatment arms to explore preliminary metrics of the intervention's effects on mood, smoking, and physical activity. By convention, participants who were missing smoking data were coded as smokers. For other outcomes, we completed a complete case analysis since retention rates were so high and similar across groups. Descriptive statistics were used to characterize the study sample, treatment utilization, and program ratings. Change from baseline to follow-up was used as the outcome to assess the intervention's impact on depressive symptom scores and average weekly duration of moderate and vigorous physical activity. Group differences were compared using chi-squares and likelihood ratio tests for binary outcomes and t-tests for continuous variables. Confidence intervals for odds ratios were based on the profile likelihood.

## Results

### Participant Characteristics

Based on the eligibility screening data, 79% of participants reported three 'at risk' behaviors based on their current smoking, moderate or greater depressive symptoms, and self-reports of low physical activity. The remaining participants were 'at risk' based on their smoking and depression, but endorsed moderate levels of physical activity, which they were encouraged to maintain.

Baseline characteristics are presented in Table 1. Participants were predominantly female (67.3%) and white (63.5%), smoked about half a pack a day, were moderately nicotine dependent, and had moderate levels of depressive symptoms. Self-reported motivation for wanting help managing one's mood, quitting smoking, and being physically active were generally high and self-reported self-efficacy for changing each behavior was moderate. No statistically significant baseline differences were observed (all p's ≥.05).

**Table 1:** Baseline participant characteristics

	Overall (n = 52) n (%)	Usual Care (n = 25) n (%)	Experimental (n = 27) n (%)	*p*
Female	35 (67%)	17 (63%)	18 (72%)	0.49
White	33 (64%)	20 (74%)	16 (64%)	0.44
Married/living with partner	26 (50%)	14 (52%)	12 (48%)	0.78
Education				0.63
College degree or greater	15 (29%)	7 (26%)	8 (32%)	
Smoking stage of change				0.85
Precontemplation	15 (29%)	9 (33%)	6 (24%)	
Contemplation	21 (40%)	9 (33%)	12 (48%)	
Preparation	16 (31%)	9 (33%)	7 (28%)	
Taking antidepressant	35 (67%)	17 (62%)	18 (72%)	0.49

	Mean (SD ^a^)	Mean (SD ^a^)	Mean (SD ^a^)	*p*

Age	44.5 (11.8)	45.4 (11.8)	43.5 (11.8)	0.57
Nicotine Dependence ^b^	2.37 (2.06)	2.26 (2.14)	2.46 (2.02)	0.74
Cigarettes/day	10.6 (7.2)	11.1 (7.5)	10.0 (6.9)	0.61
Depressive symptom score ^c^	1.77 (0.61)	1.68 (0.64)	1.87 (0.57)	0.26
Physical activity				
Days/week walk for exercise	1.52 (1.82)	1.48 (1.97)	1.56 (1.69)	0.88
Vigorous minutes/week	42.5 (142.3)	73.3 (193)	9.2 (18)	0.26
Moderate minutes/week	127.4 (295.4)	137.4 (270)	116.6 (326)	0.80
Walking per week (minutes)	257.3 (451.0)	264.8 (494)	249.2 (409)	0.90
Walking per week (hours)	4.3 (7.5)	4.4 (8.2)	4.2 (6.8)	0.90
Sitting per week (minutes)	510.0 (231.4)	546.7 (225)	470.4 (236)	0.24
Self-efficacy ^d^				
Quitting smoking	6.48 (2.26)	6.15 (2.37)	6.84 (2.14)	0.27
Regular physical activity	6.49 (1.74)	6.33 (1.49)	6.84 (2.14)	0.50
Managing depression	5.88 (1.37)	6.04 (1.21)	5.7 (1.52)	0.37
Motivation ^d^				
Quitting smoking	8.23 (2.14)	8.19 (2.47)	8.28 (1.77)	0.87
Physical Activity	7.98 (1.51)	8.11 (1.4)	7.84 (1.62)	0.52
Getting help managing depression	8.16 (2.26)	8.04 (2.58)	8.29 (1.88)	0.69

^a ^Standard deviation (SD).

^b ^Nicotine dependence assessed using FTND. Scores range from 0 to 10.

^c ^Depressive symptoms assessed via Hopkins SCL-D. Scores range from 0 to 4.

^d ^Scores range from 0 'not at all' to 10 'extremely'.

Despite attempting to screen out participants with physical limitations, two participants were randomized to treatment that had conditions (blindness and cerebral palsy) which were not disclosed during screening and which limited participation in aspects of the intervention. These individuals were retained in the pilot intervention and data analyses because we could not rule out the possibility that similar individuals had been included in the control condition.

### Intervention Participation

Experimental participants were eligible for up to 12 total counseling calls, scheduled during a six month period and ending before completion of the final assessment call. Thirty percent completed all 12 calls, 56% completed ≥ 10 calls, and 67% completed ≥ 6 calls. Average call duration was 30 minutes. Despite the longer intervention program, participation rates were similar to those observed in prior implementations of the cognitive-behavioral depression program [39] and the motivational smoking cessation program [40] that were used as the basis of the revised, multiple-risk factor counseling program.

### Intervention Program Ratings

Experimental participants rated each of the Step Up program components as at least moderately helpful, although aspects of the program that involved the health coach and the calls were rated more highly than the program workbook or weekly experiments (Table 2). Participants were also satisfied with the content (mean = 4.35, SD = 1.03), length (mean = 4.17, SD = 1.15), and number of the counseling calls (mean = 4.17, SD = 1.03). The findings suggest that no program elements should obviously be removed or re-designed.

**Table 2:** Experimental participants' ratings of Step Up components

Program Component	How Helpful Mean (SD ^a^)
Counseling calls	4.30 (1.47)
Step Up workbook	3.35 (1.58)
Weekly homework experiments	3.52 (1.44)
The health coach	4.48 (1.04)
Speaking with someone about mood	4.43 (0.95)
Speaking with someone about physical activity	4.17 (0.94)
Speaking with someone about smoking	3.91 (1.20)

Note: Components were rated from 1 (not at all helpful) to 5 (extremely helpful).

^a ^Standard deviation (SD)

### Impact on Mood, Smoking, and Physical Activity

**Figure 2:**
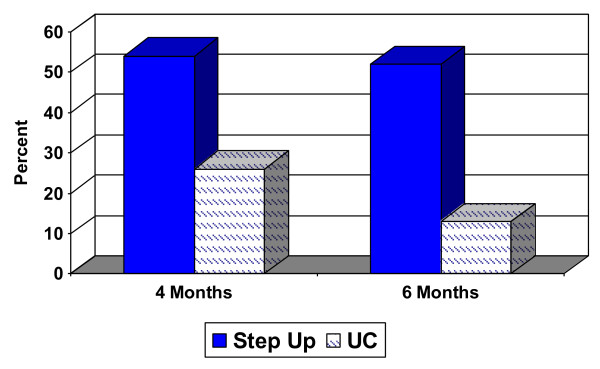
**Proportion with a clinically significant (50%) reduction in depressive symptom score**. Note: Proportion with a clinically significant reduction in SCL-D depression scores from baseline to follow-up between experimental (Step Up) and usual care (UC) controls: 54% vs. 26%, OR = 3.35, 95% CI [1.01- 12.10], *p *= 0.05 at 4 months and 52% vs. 13%, OR = 7.27, 95% CI [1.85 - 37.30], *p *= 0.004 at 6 months.

**Figure 3:**
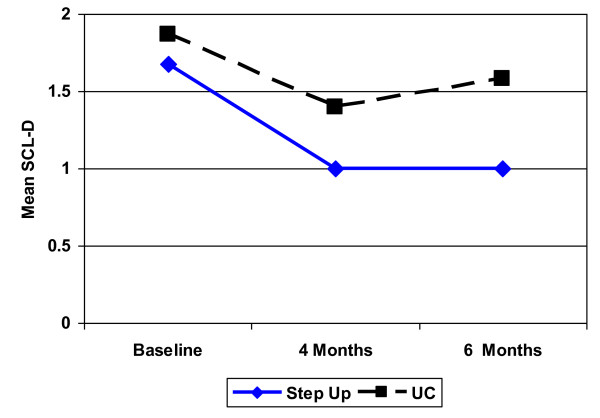
**Change in mean depression score over time**. Note: Mean SCL-D depression score among Step Up and usual care (UC) controls.

Significantly more experimental participants reported a clinically significant improvement (50% reduction) in their baseline SCL depression score at four months (54% vs. 26%, OR = 3.35, 95% CI [1.01- 12.10], *p *= 0.05) and 6 months (52% vs. 13%, OR = 7.27, 95% CI [1.85 - 37.30], *p *= 0.004) (Figure 2). The mean SCL-D score at baseline and each follow-up are depicted in Figure 3 and mean change over time is presented in Table 3. Although group differences in SCL scores were not statistically significant, the observed trend was in the expected direction and the 95% CI's suggest that clinically important intervention effects cannot be ruled out. At the same time, however, we cannot rule out the possibility of no intervention effect in a larger sample. We did not collect detailed information about antidepressant dose or duration over the course of treatment, but at 6 month follow-up 2 of 18 experimental participants had stopped taking their medication; follow-up data was not available for a third person who had been taking medication at baseline. Two of the 17 usual care participants also stopped taking their medication by follow-up, but 2 other participants had begun taking an antidepressant. Thus, more usual care participants were on medication at follow-up. It is not clear what impact medication use had on mood outcomes, but it appears unlikely that the clinically and statistically significant improvement observed in the experimental group was due to greater overall antidepressant use in this group.

**Table 3:** Change in depression, smoking, and physical activity outcomes

	Mean Change ^a^			95% CI ^b ^for Difference		
	Experimental	Control	Difference in Means	Lower	Upper	*p*
Mean change in SCL-D ^c^:						
Month 4	-0.70	-0.49	-0.21	-0.61	0.18	0.28
Month 6	-0.68	-0.32	-0.36	-0.78	0.06	0.09
Mean change cigs/day ^d^:						
Month 4	-3.2	-1.8	-1.3	-4.1	1.4	0.34
Month 6	-2.1	-2.4	0.3	-2.3	2.9	0.83
Mean change in Days Walked/week:						
Month 4	1.17	0.13	1.04	-0.17	2.24	0.09
Month 6	1.13	0.74	0.39	-1.21	1.99	0.62
Mean change in minutes/week of Moderate PA ^e^:						
Month 4	71	38	33	-220	285	0.80
Month 6	121	96	25	-251	299	0.86
Mean change in minutes/week of Vigorous PA ^e^:						
Month 4	1	78	-77	-190	36	0.18
Month 6	3	60	-57	-179	65	0.35
	
	**Experimental**	**Control**	**Odds Ratio**	**95% CI**		***p***
	
Percent made Quit Attempt						
Month 4	67%	48%	2.18	0.68	7.30	0.19
Month 6	83%	65%	2.67	0.70	11.60	0.15
Percent abstinent:						
Month 4	15%	16%	0.91	0.19	4.31	0.91
Month 6	11%	20%	0.50	0.90	2.29	0.37

^a ^Mean change from baseline to follow-up.

^b ^Confidence interval (CI).

^c ^Symptom Checklist depression score (SCL-D)

^d ^Cigarettes smoked per day

^e ^Physical activity (PA)

Impact on smoking and physical activity measures are also presented in Table 3. Several of the observed measures appeared to trend in the expected direction favoring more positive behavior change in the experimental intervention. While none of these comparisons were statistically significant, the 95% CI's for each outcome are wide, meaning that we cannot rule out the possibility of a meaningful group difference in a larger sample. At the same time, we also cannot rule out the possibility of no intervention effect in a larger sample.

## Discussion

The Step Up pilot trial was designed to determine the acceptability and feasibility of a phone-based, cognitive-behavioral, MRF intervention targeted to depressed smokers. We also sought insight into how to best design and implement this type of treatment program, to guide a future randomized effectiveness trial and to inform future work in this area. Our results support the acceptability and feasibility of the Step Up program. Two-thirds of people who were eligible for the trial enrolled, two-thirds of enrollees completed six or more counseling calls, and a third completed all twelve calls. Participation may have been greater if the treatment window were not truncated at six months to accommodate the pilot timeline. Participants were also engaged during the calls, as evidenced by the average call duration (30 minutes). They rated their satisfaction with the program as high and each of the program components as moderately helpful or greater.

As a pilot trial, the study was not powered to detect clinically significant outcomes, so caution must be used in interpreting the results, but two important findings are worth noting. First, mood significantly improved (statistically and clinically) in the experimental group. This is not surprising since the depression module was largely adapted from a prior empirically-validated program [39], but positive effects were maintained in the absence of the medication management component included in the original treatment design, and the intervention's effect on mood did not appear to be negatively impacted by the inclusion of counseling for physical activity or smoking cessation. Second, several physical activity and smoking outcomes appeared to trend in the expected direction. More intervention participants attempted to stop smoking, and this group appeared to have a greater initial reduction in their daily smoking level. They also reported walking more days for exercise each week and more positive improvements in the amount of moderate physical activity they engaged in. At the same time, however, usual care participants reported a greater increase in vigorous physical activity. It is unclear why experimental participants would report greater moderate physical activity and controls report increased vigorous physical activity, but each of the study findings must be viewed with caution. Positive changes could reflect response biases rather than true behavioral changes, since they are based on self-report in a small sample. Moreover, given the small number of participants, one cannot conclude that similar effects would be observed in a larger sample. To truly evaluate the impact of the Step Up intervention on the behavioral outcomes of interest, a larger randomized trial is needed.

This study provides insight into several important design considerations for future research. First, while making a quit attempt is an important indicator of motivation and behavior change, smoking cessation is needed to ultimately reduce disease risk. We cannot draw conclusions about the relative impact of the Step Up intervention on cessation based on our pilot data, but based on our knowledge of the actual counseling call discussions, we are unsure whether the current intervention would have a meaningful impact on cessation in a larger trial. We intentionally targeted all smokers - regardless of their interest in quitting - to see if we could engage individuals who might not otherwise be reached by conventional cessation treatment programs. In this respect, we were successful, but the intervention may not be intensive enough to promote and support quitting among those with no interest in cessation. In the future, it is worth considering increasing the intensity of the cessation treatment (i.e., longer duration, pharmacotherapy), targeting smokers with some interest in quitting, or both. More intensive treatment and pharmacotherapy have both been shown to enhance quit rates [45], so these enhancements should increase the likelihood that participants will successfully quit smoking.

The findings from this study also provide evidence of the acceptability of targeting multiple risk behaviors concurrently. Physical activity is a natural component of behavioral activation, which is an effective form of depression intervention [46]. Thus, integrating both behaviors into behavioral activation experiments was relatively straightforward and well received by participants. Moreover, participants were receptive to the integrated focus on smoking, which included weekly self-monitoring and self-efficacy building behavioral experiments such as practicing how to delay smoking in response to urges, changing their smoking environment, and making practice quit attempts. Furthermore, the current design allowed people to self-select to what extent they chose to attempt sequential or concurrent behavior change, since they ultimately chose which behavioral experiments to try. Thus, we cannot conclude that our concurrent method is truly preferable to sequential intervention, but this research provides a basis to recommend a similar intervention format to others.

We also found it better to focus on increasing self-directed physical activity than to require participation in a group-based, structured walking program. The walking group program was designed to provide social support, which is an important therapeutic component of CBT, but the increased social anxiety and avoidance that are often characteristic of depression created an insurmountable barrier to participation in the walking groups. According to participants, their guilt about not participating in the groups, in turn, adversely influenced their participation in the pre-pilot counseling program. Thus, despite the sound theoretical argument for group-based physical activity for people with depression, future interventions targeting this population may be better served by promoting personalized, self-paced activity.

Finally, it is worth noting that only 13% of those contacted were eligible for participation in this trial, even with our more lenient physical activity criterion which screened out highly active people but retained those who needed to improve or maintain their activity level. While this rate is not expected to generalize to other settings or recruitment methods, it does raise an important issue. Requiring criterion performance on multiple target behaviors can significantly reduce the number of potentially eligible MRF program candidates. Those eligible represent a high-risk, high-need population, but from a pragmatic standpoint, more people would be eligible and efforts to change may be more successful if people with three or more risk behaviors are not targeted. Requiring criterion performance on any two behaviors would make this type of program more generalizable, but doing so opens up important questions about how to measure the success of the program if not all participants are attempting to change the same behaviors. In this event, it would be important to measure success using an index of change across behaviors, such as that proposed by Prochaska and colleagues [6].

## Conclusions

These results add to the growing literature on MRF interventions. While there is some evidence that programs targeting more than one behavior at a time can be effective [4,32,47], the data are still inconclusive as to whether a combined intervention approach is truly a more effective way to change behavior or reduce disease risk [4,48]. Moreover, the relative infancy of the MRF intervention field means that there are important methodological questions which must be addressed before we can reach consensus about the optimal methods for evaluating these programs.

The results of this pilot trial demonstrate that it is both acceptable to participants and feasible from an organizational perspective to address multiple risk behaviors in a single phone-based counseling program. Moreover, positive trends in behavior change were observed. The results of this formative research provide important insight to inform and enhance future work in the field of multiple risk factor interventions.

## Competing interests

The authors declare that they have no competing interests.

## Authors' contributions

JM conceived of the study, secured the research funding, created the intervention and assessment materials, supervised study interventionists and participated in the analyses and preparation of this manuscript. SLC and EJL assisted in the conception of the intervention design and program materials, supervision of study interventionists, interpretation of the data analyses, and preparation of this manuscript. JR provided oversight of the clinical trial and assisted in the preparation of this manuscript. KR assisted in delivering the study intervention, interpretation of the research findings, and preparation of this manuscript. LG assisted in the original study design, conducted the statistical analyses, and assisted in the interpretation of study results and the preparation of the manuscript. All authors read and approved the final manuscript.

## Pre-publication history

The pre-publication history for this paper can be accessed here:

http://www.biomedcentral.com/1471-2458/11/167/prepub
